# HARQ Performance Limits for Free-Space Optical Communication Systems

**DOI:** 10.3390/e28010016

**Published:** 2025-12-23

**Authors:** Giorgio Taricco

**Affiliations:** Department of Electronics and Telecommunications, Politecnico di Torino, 10129 Turin, Italy; giorgio.taricco@polito.it

**Keywords:** free-space optical (FSO) communications, HARQ, finite blocklength, channel dispersion, OOK

## Abstract

Free-space optical (FSO) communications represent an attractive technology for future high-capacity wireless and satellite networks, offering multi-Gbps data rates, unlicensed spectrum, and built-in physical-layer security. However, their performance is severely affected by atmospheric turbulence, misalignment errors, and noise, which limit reliability and throughput. Hybrid automatic repeat request (HARQ) protocols provide a powerful mechanism to mitigate such impairments by combining forward error correction with retransmissions. In this paper, we investigate the fundamental performance limits of HARQ applied to FSO systems employing On–Off Keying (OOK) modulation. Using information-theoretic tools, we characterize the achievable rate and the finite-blocklength performance by resorting to channel dispersion, which plays a crucial role in quantifying rate–reliability tradeoffs. We further examine the interaction between HARQ retransmissions, turbulence-induced fading, and feedback delay, providing insights into the design of low-latency, high-reliability optical links. This analysis highlights how HARQ improves the robustness of OOK-based FSO systems and provides guidelines for parameter selection in next-generation space and terrestrial optical networks.

## 1. Introduction

The demand for ultra-high data rates and reliable connectivity has increased with the spreading of data-intensive applications, such as virtual/augmented reality, autonomous driving, and satellite-based internet. Meanwhile, radio-frequency (RF) systems are increasingly constrained by spectrum and interference limitations. This motivates research into alternative technologies. Free-space optical (FSO) communications have emerged as a valid alternative to RF systems due to their abundant unlicensed spectrum, high directionality, and immunity to electromagnetic interference [1,2]. Additionally, FSO links support multi-Gbps data rates over long distances, offer low power consumption, and provide inherent physical-layer security because of the highly focused transmitted signal. Within the broader context of wireless and satellite networks, recent works on space–air–ground-integrated architectures, such as [3], further highlight the importance of accurate channel modeling and resource allocation for future high-capacity optical and hybrid systems.

Despite the advantages of FSO communications, optical links are susceptible to atmospheric impairments, including turbulence, absorption, scattering, pointing errors, and cloud blockage. These effects induce fading and outages that reduce both achievable throughput and reliability [1,4]. To combat these impairments, a variety of physical-layer solutions (such as adaptive coding, aperture averaging, diversity relaying, concatenated coding, and interleaving) and link-layer techniques (such as error correction and retransmissions) have been investigated. Among them, Automatic Repeat reQuest (ARQ) and Hybrid ARQ (HARQ) protocols provide powerful mechanisms to mitigate such impairments by combining forward error correction (FEC) with retransmissions, thereby exploiting both coding gain and retransmission diversity.

Another key design choice is the modulation format. While advanced schemes such as pulse-position modulation (PPM) or coherent modulation offer higher rates, On–Off Keying (OOK) with direct detection remains appealing for its simplicity, cost-effectiveness, and compatibility with commercial optical components [1]. In turbulence-limited FSO systems, OOK combined with HARQ represents a pragmatic balance between complexity and performance, especially in scenarios where hardware constraints, standardization aspects, and implementation maturity favor intensity-modulation/direct-detection solutions over more sophisticated coherent receivers. Understanding the fundamental rate limits of these systems is crucial to support the design of next-generation terrestrial and space optical networks.

Most published works on HARQ for FSO rely on classical information-theoretic metrics, such as outage probability, ergodic capacity, and long-term throughput. However, emerging applications, such as Direct-To-Earth (DTE) LEO downlinks, require stringent latency and reliability constraints to be guaranteed in addition to spectral efficiency. This has motivated the usage of finite-blocklength information theory concepts, which extend the achievable-rate analysis beyond Shannon’s asymptotic regime by incorporating the channel dispersion as a key parameter [5,6,7]. This framework enables accurate performance characterization at short blocklengths, quantifying the trade-off between achievable rate, latency, and error probability, and is particularly relevant when HARQ retransmissions interact with coding and feedback delays.

In this paper, we analyze the performance limits of HARQ in OOK-based FSO systems within the finite-blocklength regime. We consider a LEO direct-to-Earth optical downlink at λ=1550 nm, where the channel is modeled through realistic power vectors representative of turbulence-induced fading. Using information-theoretic tools, the mutual information, capacity, and channel dispersion of the underlying OOK channel under shot-noise dominance are characterized and incorporated into a queue-aware HARQ analysis. The focus is on HARQ-I and HARQ-II as representative baseline protocols: the key difference is that HARQ-II explicitly accounts for soft combining of retransmissions. The delay analysis is based on the implementation of the actual retransmission queue, rather than on approximate latency models such as those used in [7], thereby providing a tighter connection between information-theoretic performance limits and packet-level quality-of-service metrics.

The scenario considered corresponds to a *turbulence-limited*, *clear-sky*, *well-aligned FSO regime*, which is representative of controlled experimental testbeds and scheduled LEO–ground contacts where (i) cloud-blocked intervals are excluded through site-diversity or weather filtering, and (ii) closed-loop tracking reduces residual pointing jitter well below the beam divergence. Specifically, these operating conditions do not cover the case of impaired weather (e.g., fog/haze) or misaligned terminals. Within this framework, the main contributions of this work can be summarized as follows:

- Information-theoretic characterization (capacity and dispersion) of an OOK-based FSO channel tailored to realistic LEO downlink conditions.
- Integration of these finite-blocklength results into a queuing-based HARQ performance analysis that jointly captures frame error rate (FER), throughput, and end-to-end delay under turbulence-induced fading.
- Quantification of the impact of packet length and retransmission limits on the achievable reliability–latency trade-offs, and highlight the regimes where combining HARQ-II yields significant throughput gains over HARQ-I while offering limited delay improvements due to queuing and feedback constraints.

To the best of the author’s knowledge, this is the first work to combine finite-blocklength information theory, realistic OOK-based LEO FSO channel modeling via power vectors, and HARQ-II with explicit retransmission-queue modeling in order to assess fundamental performance limits in terms of FER, throughput, and latency.

### 1.1. Related Literature Results

This subsection organizes the related literature into three main categories: (i) FSO channel impairment mitigation; (ii) HARQ protocol optimization for FSO and related systems; (iii) applications of finite-blocklength information theory.

#### 1.1.1. FSO Channel Impairment Mitigation

A comprehensive overview of FSO channel characteristics and impairment-mitigation techniques is provided in [1,2,8,9,10,11,12]. These works discuss the main atmospheric effects, including turbulence, absorption, scattering, pointing errors, and cloud blockage. They also survey several mitigation strategies such as aperture averaging, spatial and temporal diversity, adaptive optics, advanced modulation and coding, and hybrid RF/FSO architectures. In this framework, turbulence-induced scintillation is often modeled using log-normal, Gamma–Gamma, or more sophisticated statistical models [13,14], and a number of studies focus on level-crossing rate, average outage duration, and fading coherence time to characterize link reliability and diversity potential [15,16,17].

More recently, attention has shifted towards realistic channel representations tailored to specific deployment scenarios. In this spirit, Giggenbach et al. proposed the PVGeT power-vector generation tool and associated reference power vectors for the optical LEO downlink channel [18,19]. The PVGeT power-vector generation tool provides time-series samples representing the combined impact of turbulence and pointing jitter under typical conditions. This modeling has also been addressed in other works, such as [20], where ground-to-UAV sub-terahertz channel measurements and modeling are used. These efforts are conceptually aligned with the modeling approach used in this paper, where consolidated power vectors allow us to bridge the gap between abstract fading models and simulation-based performance evaluation.

#### 1.1.2. HARQ Protocol Optimization for FSO Systems

The application of ARQ and HARQ to FSO links has been investigated in several works. Early studies such as [21] examined hybrid ARQ schemes for FSO communications through a turbulent atmosphere, demonstrating the potential of retransmissions to improve link robustness without excessive redundancy. Subsequent works focused on more refined HARQ variants and on a combination of coding and diversity techniques. For instance, Aghajanzadeh and Uysal [22] provided an information-theoretic analysis of HARQ protocols in coherent FSO systems, highlighting the gains obtainable by combining FEC with retransmissions in turbulence-limited channels. Zedini et al. [23] analyzed IR-HARQ and code-combining schemes over FSO channels with pointing errors, deriving closed-form performance expressions and showing that incremental redundancy can approach capacity under favorable conditions. Makki et al. [24] studied RF–FSO links with and without HARQ, comparing different protocol configurations and shedding light on design trade-offs across heterogeneous links. Other works, such as [4,25,26], further explored HARQ performance over FSO channels with atmospheric fading, pointing errors, or rate adaptation, often under asymptotic assumptions in terms of blocklength or averaging.

On the experimental side, Schieler et al. [27] illustrated the practical benefits of retransmission-based reliability enhancement. Related contributions addressing coding and synchronization for space optical communications [28,29,30,31,32,33,34,35] provide complementary insights into how HARQ can be integrated with advanced coding and interleaving architectures.

Most of these works, however, adopt asymptotic information-theoretic metrics (e.g., outage probability and ergodic capacity) without explicitly incorporating finite-blocklength effects or detailed retransmission queuing behavior. Moreover, while HARQ-II combining and incremental-redundancy schemes are often analyzed, the explicit combination of realistic OOK FSO channels, finite-blocklength analysis, and queue-based delay modeling has not been addressed to the best of the author’s knowledge.

#### 1.1.3. Finite Blocklength Theory and HARQ

Finite-blocklength information theory, as developed in [5] and extended in the recent book [6], provides refined performance bounds that account for channel dispersion and quantify the rate penalty incurred at moderate blocklengths. These tools have been applied to a variety of wireless communication problems, including latency-constrained and ultra-reliable scenarios. In the context of HARQ, Makki et al. [7] proposed the so-called *fast HARQ* protocol, analyzing its performance over finite-blocklength codes and deriving low-latency reliability trade-offs. Their analysis is based on approximate models for the retransmission delay and does not target optical OOK FSO systems or realistic LEO downlink power-vector channels.

In contrast, the present work adopts the finite-blocklength framework to characterize the mutual information and channel dispersion of an OOK-based FSO channel with shot-noise dominance, and then embeds these quantities into an explicit queuing model that accounts for retransmissions and feedback delay. The retransmission delay is obtained from the implementation of the actual queue, rather than from simplified analytic approximations, which allows for a more faithful representation of latency and its dependence on system parameters. In this sense, the paper complements and extends the existing finite-blocklength HARQ literature by focusing on OOK-based FSO links, leveraging realistic power-vector channels, and emphasizing the joint analysis of FER, throughput, and end-to-end delay under turbulence-limited, clear-sky operating conditions.

## 2. System Model

An FSO link transmits data through a laser beam between a satellite and a ground station. This link exploits the wide optical bandwidth to achieve very high rates, but its performance is degraded by atmospheric turbulence.

### 2.1. General Features

The system comprises three main blocks: *transmitter*, *channel*, and *receiver*. At the transmitter, a laser generates the optical carrier, which is modulated (e.g., ASK, PSK, QAM), shaped by transmit optics, and aligned by the pointing, acquisition, and tracking (PAT) subsystem [8,9,11,12,13,14].

During propagation, the beam is subject to *absorption and scattering* by water vapor, aerosols, and dust, as well as *turbulence* from refractive index fluctuations, producing beam wander, scintillation, and wavefront distortion [14]. Turbulence can be modeled using Kolmogorov statistics, with metrics such as Fried’s parameter $r_{0}$ and Rytov variance.

At the receiver, a large-aperture optical antenna collects the signal, which is demodulated and possibly corrected with adaptive optics and digital signal processing.

The link budget accounts for power, aperture gains, propagation loss, turbulence, pointing, and receiver sensitivity. The received power is

(1) $$P_{r}=P_{t}G_{t}G_{r}\eta_{t}\eta_{r}\frac{A_{r}}{L^{2}}T_{\mathrm{atm}}e^{-\sigma_{\mathrm{tur}}L},$$

where $P_{t}$ is transmitted power, $A_{r}$ is the aperture area, *L* is the link distance, $T_{\mathrm{atm}}$ the atmospheric transmission factor (characterizing how much of the transmitted optical power is not absorbed or scattered by the atmosphere as the beam propagates through it), and $\sigma_{\mathrm{tur}}$ is the turbulence attenuation coefficient. Turbulence statistics are often modeled by log-normal, Gamma–Gamma, or Fokker–Planck distributions, but in this work we are using consolidated power vector samples collected by [18]. In [18], the test vectors are generated using the PVGeT simulation tool, which models atmospheric turbulence and pointing jitter as independent fading processes. Lognormal scintillation vectors are produced by filtering Gaussian samples with a Butterworth low-pass filter and applying a nonlinear transformation to match the desired Power Scintillation Index. Pointing-jitter fading vectors are created from Gaussian angular jitter samples, spectrally shaped and converted into radial beam wander, yielding a beta-distributed power loss. The final test vector is obtained by multiplying the scintillation and pointing components, producing realistic received-power time series that replicate measured FSO link statistics.

It is worth mentioning that the characterization of fading and power vector-based modeling has also been addressed by Li et al. [20], who provided an empirical framework for modeling atmospheric channels and fading statistics. This study is conceptually aligned with the modeling approach used here.

It is also worth noting, for later use in the channel model of Section 2.3, that PVGeT provides received-power samples. These samples are converted into the dimensionless fading variable *H* through $H_{n}=\sqrt{P_{r}\left[n\right]/E\left[P_{r}\right]}$, so that the model $Y=HX+\sigma_{X}Z$ and the equivalent-SNR Definition (11) remain consistent.

### 2.2. Impact of Fog and Pointing-Induced Fading

In this work, we focus on a *turbulence-limited* operating regime, which is a special case of a more general model where the received optical power can be expressed according to the following expression:

(2) $$P_{r}=P_{t}\;L_{\mathrm{fog}}\;L_{\mathrm{point}}\;h_{\mathrm{turb}}.$$

Here, $P_{t}$ is the transmitted optical power, $h_{\mathrm{turb}}$ denotes the turbulence-induced fading coefficient, $L_{\mathrm{fog}}\in \left[0,1\right]$ accounts for deterministic attenuation due to fog or haze, and $L_{\mathrm{point}}\in \left[0,1\right]$ represents the power loss from residual pointing errors. This factorization is consistent with the link-budget modeling used in [36,37]. The former addresses geometric losses, weather-dependent extinction, and turbulence-induced fading as separable multiplicative terms, and the latter characterizes the impact of visibility-based fog-attenuation. Specifically, fog attenuation is accounted for by the exponential Beer–Lambert law, $L_{\mathrm{fog}}=\exp\left(-\alpha_{\mathrm{fog}}d\right)$, where $\alpha_{\mathrm{fog}}$ is the specific attenuation and *d* the link distance. This representation is employed both in [36], which compares several empirical fog-attenuation models (Kruse, Kim, Al Naboulsi, and the unified model proposed therein), and in [37], which adopts the Kruse visibility law to map long-term meteorological data to wavelength-dependent attenuation. In particular, the measurements compiled in [36] indicate that $\alpha_{\mathrm{fog}}$ may reach hundreds of dB/km under dense fog, whereas the multi-year analysis in [37] shows that for typical visibilities above 3–5 km, the attenuation at 1550nm can be well below 1dB/km, suggesting that $L_{\mathrm{fog}}\approx 1$ is a reasonable approximation in such environments.

Pointing-induced loss $L_{\mathrm{point}}$ can likewise be modeled as a multiplicative factor whose statistics depend on the beam divergence, aperture diameters, and jitter variance. Although [36] does not provide a full probabilistic model for pointing errors, it quantifies the pronounced sensitivity of received power to geometric alignment, particularly for long links and narrow-beam transmitters. This motivates modeling $L_{\mathrm{point}}$ as a slowly varying or random gain independent of atmospheric turbulence. For the operational conditions studied in [37] (namely, moderate visibilities and negligible cloud blockage during the measurement period) pointing-induced effects were not a limiting factor, further supporting the regime $L_{\mathrm{point}}\approx 1$ under well-aligned, closed-loop tracking.

In this paper, we restrict attention to scenarios in which both fog attenuation and pointing loss remain small with high probability, so that turbulence-induced fading dominates the random power fluctuations. Such conditions arise in clear-sky or lightly hazy propagation, in moderate-to-high elevation passes, and in systems where the pointing jitter is small relative to the beam divergence. These assumptions agree with the measurement-based findings of [37], which report low fog-induced attenuation for most visibility conditions, and with the discussion in [36] showing that only for low-visibility fog events does extinction dominate the link budget.

Outside this parameter regime (for example, under dense fog conditions where $\alpha_{\mathrm{fog}}$ becomes large [36], or in the presence of significant misalignment), a joint turbulence–fog-pointing model becomes necessary, and link-layer mechanisms such as HARQ would need to be re-optimized accordingly. Developing such a joint framework, including the incorporation of cloud and visibility statistics as characterized in [37] and the geometric sensitivity highlighted in [36], is left for future work.

### 2.3. Channel Model

The FSO communication system considered represents a Low Earth Orbit (LEO) Direct-to-Earth (DTE) link at λ=1550 nm, using OOK modulation with rates from a few Mbps up to 10 Gbps [29,38,39]. The analysis is restricted to clear-sky, sufficiently well aligned LEO–ground links in which cloud attenuation and residual pointing errors are negligible compared to turbulence-induced fading. These assumptions are representative of high-elevation satellite passes and good weather conditions, as encountered in dedicated FSO testbeds and scheduled contact windows. Turbulence is the dominant impairment [16,17], acting as a slow fading process mitigated by time diversity. Shot noise from the APD is modeled as Gaussian with variance dependent on the received power.

Dropping the time index for the sake of simplicity, the received signal follows the power-observation model:

(3) $$\begin{array}{c} Y=HX+\sigma_{X}Z, \end{array}$$

with X∈{0,1}, fading power gain *H*, Z∼N(0,1), and

(4) $$\begin{array}{c} \sigma_{x}^{2}=\left\{\begin{array}{cc} \frac{\sigma_{t}^{2}}{2R_{s}{\left(\nu_{s}qM\right)}^{2}}, & x=0\\ \sigma_{0}^{2}+\frac{FH}{\nu_{s}}, & x=1 \end{array}\right. \end{array}$$

where $\nu_{s}$ is the average number of photons per symbol, $R_{s}$ is the symbol rate, *q* is the electron charge, *M* is the APD multiplication factor, *F* is the excess noise factor, and $\sigma_{t}$ is the TIA noise density. The symbol rate and the TIA noise density depend on μ∈{0,…,13} according to the following equations:

(5) $$\begin{array}{cc} R_{s} & =2^{-\mu }\cdot 10^{10},\;\sigma_{t}=10^{-11}e^{-0.27\mu }. \end{array}$$

The photon information efficiency (PIE) relates to $\nu_{s}$ via

(6) $$\begin{array}{c} \mathrm{PIE}=\frac{\nu_{s}}{2R_{c}}, \end{array}$$

with code rate $R_{c}$. Under thermal-noise dominance,

(7) $$\begin{array}{c} \sigma_{0}^{2}\gg \sigma_{1}^{2}-\sigma_{0}^{2}=\frac{FH}{\nu_{s}}. \end{array}$$

**Remark** **1.**  *By normalizing the channel output as $\tilde{Y}≜Y/H$, we obtain $\tilde{Y}=X+{\tilde{\sigma }}_{X}Z$, where the effective noise variances ${\tilde{\sigma }}_{0}^{2}$ and ${\tilde{\sigma }}_{1}^{2}$ depend on the fading H only through the product $\nu_{s}H$:*

$${\tilde{\sigma }}_{0}^{2}=\frac{\sigma_{t}^{2}}{2R_{s}{\left((\nu_{s}H)qM\right)}^{2}},\;{\tilde{\sigma }}_{1}^{2}=\frac{\sigma_{t}^{2}}{2R_{s}{\left((\nu_{s}H)qM\right)}^{2}}+\frac{F}{\nu_{s}H}.$$

*Therefore, for each fading realization H, the mutual information and dispersion can be computed by reusing the H=1 formulas with the replacement $\nu_{s}\mapsto \nu_{s}H$. This observation is used in the simulations when mapping PVGeT power-vector samples to instantaneous achievable rates.*

Now, an *equivalent* SNR is defined by following the approach described by [40]. Assuming equiprobable symbols with a decision threshold θ, the symbol error probability can be written as

(8) $$P_{e}\left(\theta \right)=\frac{1}{2}Q\left(\frac{\theta }{\sigma_{0}}\right)+\frac{1}{2}Q\left(\frac{H-\theta }{\sigma_{1}}\right).$$

It is plain to see that the optimal threshold $\theta_{\mathrm{opt}}$ (minimizing the error probability) corresponds to the equalization of the arguments of the *Q* functions, so that

(9) $$\theta_{\mathrm{opt}}=\frac{H\sigma_{0}}{\sigma_{0}+\sigma_{1}}.$$

Then, the minimum error probability corresponding to $\theta_{\mathrm{opt}}$ is equalized to the error probability of binary Pulse-Amplitude Modulation (PAM) over the additive Gaussian noise channel corresponding to an equivalent SNR given by SNR:

(10) $$Q\left(\frac{H}{\sigma_{0}+\sigma_{1}}\right)=Q\left(\sqrt{\frac{\mathrm{SNR}(H)}{2}}\right)\Rightarrow \mathrm{SNR}\left(H\right)=\frac{2H^{2}}{{\left(\sigma_{0}+\sigma_{1}\right)}^{2}}.$$

Finally, normalizing the fading gain by $E[H^{2}]=1$ (this normalization is imposed for consistency with the equivalent SNR formulation in (11)), we have the equivalent SNR formula reported in ([40] Equation (7)):

(11) $$\begin{array}{c} \mathrm{SNR}=2\cdot \frac{E_{s}}{N_{0}}=2R_{c}\frac{E_{b}}{N_{0}}=\frac{2}{{\left(\sigma_{0}+\sigma_{1}\right)}^{2}} \end{array}$$

**Figure 1:**
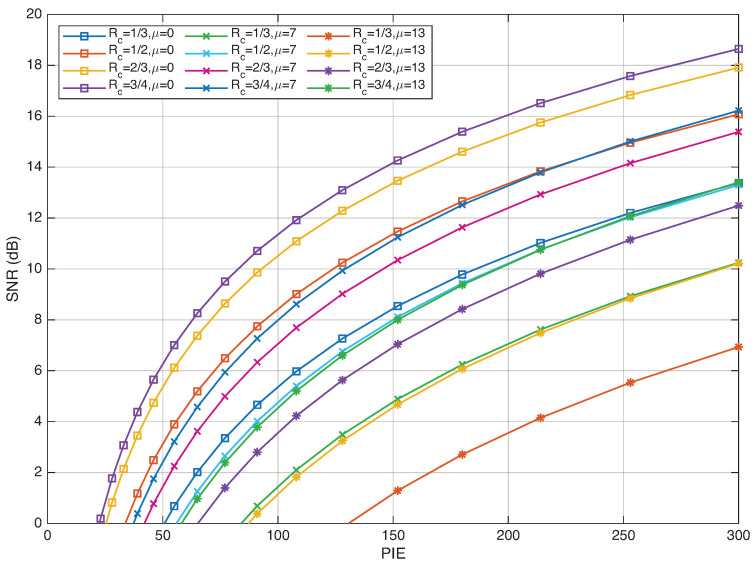
Plot of the SNR defined in (11) versus the PIE for different values of the code rate $R_{c}$.

Figure 1 illustrates the relationship between PIE and SNR for various code rates $R_{c}$.

## 3. Channel Capacity and Dispersion

According to classical information theory and its finite–blocklength generalization, two quantities characterize the achievable rate of a communication channel. The first is the *channel capacity*, which is defined as the supremum of the mutual information over all admissible input distributions under a set of physical constraints. The second is the associated *channel dispersion*, which is the variance of the information density and determines how rapidly the achievable rates depart from capacity when operating at finite blocklength [5].

Together, they provide fundamental insights into the ultimate performance limits (independent of specific coding or modulation schemes) of FSO systems impaired by turbulence and shot noise. Capacity expressions serve as critical benchmarks for evaluating system design choices, such as modulation formats, coding strategies, and HARQ protocols. Dispersion analysis provides additional insight when the system operates with limited blocklength coding. In this section, we derive the mutual information, the capacity, and the channel dispersion for the OOK-based system model considered and examine how turbulence and noise statistics affect the achievable rates.

First, we calculate the information density of the FSO channel, modeled by the equation $Y=HX+\sigma_{X}Z$, where X∈{0,1} and Z∼N(0,1). The conditional probability density function is

(12) $$\begin{array}{c} p_{Y|X}\left(y|x\right)=\frac{1}{\sqrt{2\pi }\sigma_{x}}e^{-{\left(y-Hx\right)}^{2}/\left(2\sigma_{x}^{2}\right)}. \end{array}$$

Then, assuming input probabilities $P\left(X=x\right)=p_{x}$, the received signal probability density function is given by

(13) $$\begin{array}{c} p_{Y}\left(y\right)=\frac{1}{\sqrt{2\pi }}\sum_{\overset{ˇ}{x}\in \left\{0,1\right\}}\frac{p_{\overset{ˇ}{x}}}{\sigma_{\overset{ˇ}{x}}}e^{-{\left(y-H\overset{ˇ}{x}\right)}^{2}/\left(2\sigma_{\overset{ˇ}{x}}^{2}\right)} \end{array}$$

Therefore, the information density is

(14) $$\begin{array}{cc} i(x;y) & =\log_{2}\frac{p_{Y|X}\left(y|x\right)}{p_{Y}\left(y\right)}=-\log_{2}\left\{\sigma_{x}e^{{\left(y-Hx\right)}^{2}/\left(2\sigma_{x}^{2}\right)}\sum_{\overset{ˇ}{x}\in \left\{0,1\right\}}\frac{p_{\overset{ˇ}{x}}}{\sigma_{\overset{ˇ}{x}}}e^{-{\left(y-H\overset{ˇ}{x}\right)}^{2}/\left(2\sigma_{\overset{ˇ}{x}}^{2}\right)}\right\}\\ \; & =-\log_{2}p_{x}-\log_{2}\left\{1+\rho_{x}\left(y\right)\right\}, \end{array}$$

where $\bar{x}≜1-x$ and

(15) $$\begin{array}{c} \rho_{x}\left(y\right)≜\frac{p_{\bar{x}}\sigma_{x}}{p_{x}\sigma_{\bar{x}}}e^{{\left(y-Hx\right)}^{2}/\left(2\sigma_{x}^{2}\right)-{\left(y-H\bar{x}\right)}^{2}/\left(2\sigma_{\bar{x}}^{2}\right)}. \end{array}$$

Thus, the mutual information is given by

(16) $$\begin{array}{c} I\left(X;Y\right)=H_{b}\left(p_{0}\right)-\sum_{x\in \{0,1\}}p_{x}E\left[\log_{2}\left\{1+\rho_{x}\left(Y\right)\right\}\right]. \end{array}$$

In this expression, $H_{b}\left(p\right)≜-p\log_{2}p-\left(1-p\right)\log_{2}\left(1-p\right)$ is the binary entropy function. The mutual information is found by numerical integration and the capacity is determined by maximizing the mutual information with respect to the probability $p_{0}\in \left[0,1\right]$.

In the finite blocklength regime defined in [5], the asymptotic notion of channel capacity is no longer sufficient to characterize the maximum coding rate. Instead, the key quantity is the channel dispersion *V*, defined as the variance of the information density:

(17) $$V=\mathrm{Var}\left[i(X;Y)\right].$$

The dispersion is a measure of the variability of the channel information rate. It allows us to approximate the maximum coding rate that can be achieved at block length *n* and error probability ε by the following asymptotic relation for n→∞:

(18) $$R^{*}\left(n,\varepsilon \right)=I\left(X;Y\right)-\sqrt{\frac{V}{n}}\;Q^{-1}\left(\varepsilon \right)+O\left(\frac{\log n}{n}\right).$$

Hence, the channel dispersion bridges the gap between asymptotic (in the coding length) capacity results and practical performance in realistic finite-length systems. For the channel considered, the dispersion can be obtained by following the standard approach of [5]:

(19) $$V=\mathrm{Var}\left[i\left(X;Y\right)\right]=E\left[i{\left(X;Y\right)}^{2}\right]-{\left(E[i(X;Y)]\right)}^{2}=\sum_{x\in \{0,1\}}p_{x}E\left[i{\left(x;Y\right)}^{2}\right]-I{\left(X;Y\right)}^{2}.$$

This expression can be evaluated by numerical integration.

**Remark** **2.**  *The calculation of the mutual information and of the channel dispersion require the integrals*

$$\int_{-\infty }^{\infty }{\left[-\log_{2}p_{x}-\log_{2}\left(1+\frac{p_{\bar{x}}\sigma_{x}}{p_{x}\sigma_{\bar{x}}}e^{z^{2}/2-{\left(H\left(x-\bar{x}\right)+\sigma_{x}z\right)}^{2}/\left(2\sigma_{\bar{x}}^{2}\right)}\right)\right]}^{\kappa }\frac{\exp(-z^{2}/2)}{\sqrt{2\pi }}dz,\;\kappa =1,2.$$

*A common way to approximate the Gaussian-weighted integral $I=\int_{-\infty }^{\infty }f\left(z\right)\frac{\exp(-z^{2}/2)}{\sqrt{2\pi }}dz$ is to use the n–point Gauss–Hermite quadrature rule. The integral can be numerically approximated by $I\approx \frac{1}{\sqrt{\pi }}\sum_{i=1}^{n}w_{i}f\left(\sqrt{2}\;x_{i}\right)$, where ${\left\{x_{i}\right\}}_{i=1}^{n}$ and ${\left\{w_{i}\right\}}_{i=1}^{n}$ are the Gauss–Hermite nodes and weights associated with the weight function $e^{-x^{2}}$ ([41] Table 25.10).*

### 3.1. Asymptotic Analysis

Additional insight into the system operation can be obtained by examining the limiting behavior of the information density i(x;y) in the high–SNR regime. Expanding Equation (15) with $Y=Hx+\sigma_{x}Z$ and Z∼N(0,1), one obtains the following equivalence in probability distribution:

(20) $$\begin{array}{cc} \rho_{x}\left(Y\right) & ∼\;\;\frac{p_{\bar{x}}\sigma_{x}}{p_{x}\sigma_{\bar{x}}}e^{Z^{2}/2-{\left(H+\sigma_{x}Z\right)}^{2}/\left(2\sigma_{\bar{x}}^{2}\right)}. \end{array}$$

When $\sigma_{0},\sigma_{1}↓0$, it is plain to see that

(21) $$\begin{array}{c} \rho_{x}\left(Y\right)=O\left(\frac{\sigma_{x}}{\sigma_{\bar{x}}}e^{-H^{2}/\left(2\sigma_{\bar{x}}^{2}\right)}\right)\Rightarrow i\left(x;Y\right)=-\log_{2}p_{x}+O\left(\frac{\sigma_{x}}{\sigma_{\bar{x}}}e^{-H^{2}/\left(2\sigma_{\bar{x}}^{2}\right)}\right) \end{array}$$

with probability 1. As a result,

(22) $$\begin{array}{c} I\left(X;Y\right)\to H_{b}\left(p\right),\;V\to p_{0}p_{1}{\left(\log_{2}\frac{p_{0}}{p_{1}}\right)}^{2}. \end{array}$$

Thus, with equiprobable input probabilities $p_{0}=p_{1}=\frac{1}{2}$, the mutual information converges to 1 and the channel dispersion vanishes as SNR→∞.

### 3.2. Numerical Results

**Figure 2:**
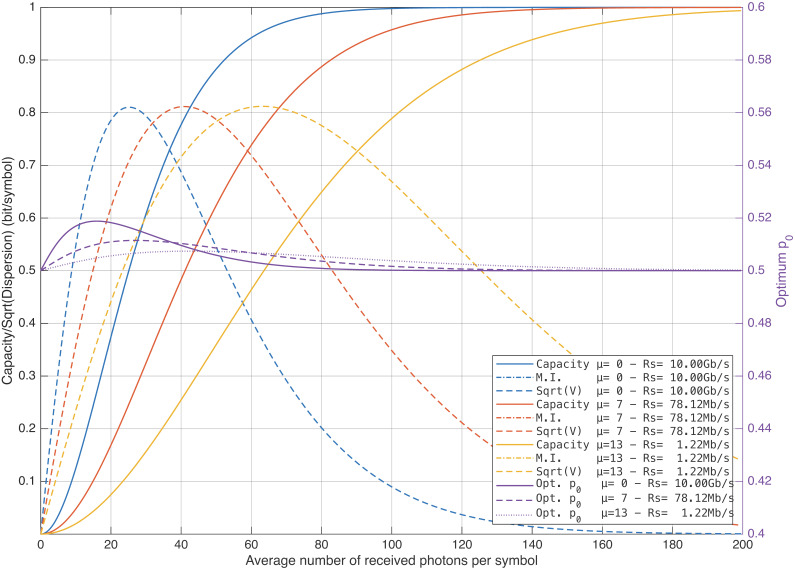
Capacity, mutual information, and square root of the channel dispersion vs. average number of received photons per symbol. Notice that there is no visible difference between the mutual information curves corresponding to equiprobable inputs and the capacity curves.

Figure 2 plots the capacity and mutual information versus $\nu_{s}$ in the no-fading case (H=1), which—see Remark 1—entails no loss of generality, since the fading channel parameters depend only on the product $\nu_{s}H$.

The figure also includes the capacity achieving probability $p_{0}^{★}$ (optimum $p_{0}$). It can be observed that $p_{0}^{★}$ is approximately equal to $\frac{1}{2}$ over the range considered, so the difference between the capacity and the mutual information corresponding to equiprobable inputs is negligible. This is positive because, usually, the encoded bit stream is approximately equiprobable. The figure shows that achieving a given rate requires a higher $\nu_{s}$ as the symbol rate decreases. Additionally, Figure 2 plots the (square root of the) dispersion versus $\nu_{s}$. It turns out that maximum dispersion is achieved when the capacity is approximately equal to $\frac{1}{2}$, which is the most commonly used coding rate. This implies that, for this coding rate, the finite-blocklength performance is maximally degraded.

**Figure 3:**
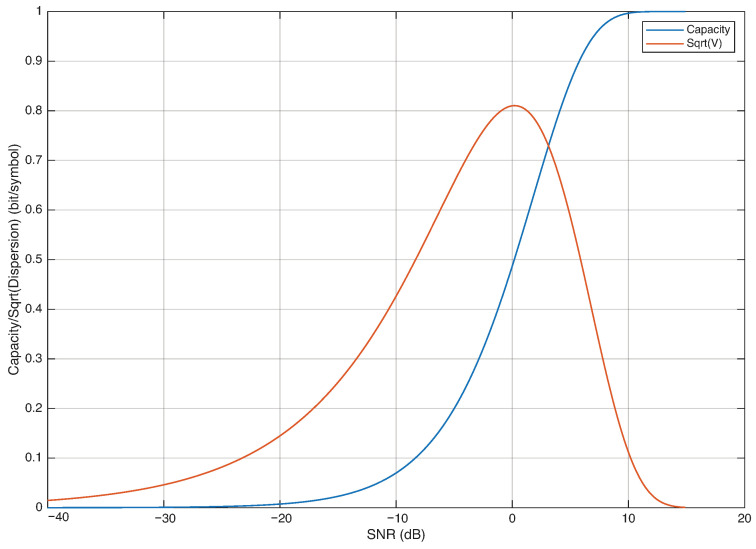
Mutual information and square root of the channel dispersion vs. SNR.

Analogous (but more compact) results are reported in Figure 3, where the mutual information and the square root of the channel dispersion are plotted versus the SNR.

## 4. Information Theoretic Analysis of HARQ

The focus of this work is on the performance of HARQ, which is a widely adopted communication protocol designed to improve the reliability of data transmission by integrating error detection mechanisms with forward error correction techniques [42]. According to this protocol, when the receiver identifies transmission errors, it resorts to a low-rate feedback channel to inform the transmitter that retransmission is required.

Different variants of HARQ determine how retransmissions are handled. In *HARQ-I*, the protocol simply consists of retransmitting the original packet until successful reception without leveraging any information from previous packet receptions, meaning each retransmission is treated independently. In contrast, *HARQ-II* combines the newly received signal corresponding to a certain data packet with previously received signals corresponding to the same packet, allowing the receiver to incrementally improve the decoding process and increase the probability of successful reception. Typically, this can be accomplished by Maximum Ratio Combining (MRC) applied to the received signal blocks corresponding to the same packet. This combination of retransmission and smart decoding enhances the overall system performance by reducing the error rate.

Due to these capabilities, HARQ is implemented in contemporary wireless communication standards, such as *LTE* and *5G*, where it plays a crucial role in boosting spectral efficiency, reducing latency, and maintaining high reliability under varying channel conditions [43,44,45].

**Remark** **3.**  *Although this paper focuses on HARQ-I and HARQ-II as representative baseline protocols, the analytical framework can be extended to more advanced schemes, such as Incremental Redundancy (IR-HARQ), Chase Combining (CC-HARQ), and adaptive HARQ strategies [43]. In IR-HARQ and CC-HARQ, the information accumulated after k transmissions can be incorporated by redefining the effective decoding metric (e.g., mutual information accumulation or combined SNR accumulation), and the corresponding decoding success probabilities can be updated accordingly in the queuing and delay analysis. Adaptive HARQ, where the number of retransmissions or redundancy levels depend on channel-state estimates, can be modeled by introducing a conditional distribution of decoding outcomes and service times. These extensions would enable a broader comparison of complexity, reliability, and latency trade-offs across modern HARQ mechanisms, and therefore constitute a promising direction for future work.*

### 4.1. System Analysis

The analysis relies on a set of assumptions regarding packet transmission and retransmission mechanisms. Transmitted packets span *G* successive time slots of duration 0.1 ms, which results in a packet duration of 0.1G ms. A *target coding rate* $R_{c}$ is imposed on the communication system, typically chosen as $\frac{1}{2}$ or $\frac{9}{10}$ depending on the desired trade-off between reliability and spectral efficiency (For the sake of space limitation, this paper focuses on rate-$\frac{1}{2}$ codes). A transmission is successful whenever the instantaneous achievable rate $R_{n}$ corresponding to the *n*-th time slot satisfies the inequality $R_{n}\geq R_{c}$ (The achievable rate $R_{n}$ corresponding to a certain value of the SNR, namely, SNR, is obtained from (16) where $\nu_{s}$ (average number of photons per symbol) is derived by solving the equation $\mathrm{SNR}=2/{\left(\sigma_{0}+\sigma_{1}\right)}^{2}$, according to the Definition (11), where $\sigma_{0}$ and $\sigma_{1}$ are characterized in (4)). Otherwise, decoding failure occurs and packet retransmission is required. The transmitter is informed of the outcome through a low-rate feedback channel, which incurs a delay of $N_{\Delta }$ symbol intervals due to processing and propagation. When G>1, the instantaneous achievable rates $R_{n}$ are replaced by the averages over *G* consecutive time slots ${\bar{R}}_{m}≜\frac{1}{G}\sum_{n=(m-1)G+1}^{mG}R_{n}$. These correspond to the achievable rates for a channel experiencing *G* different states, as in the case of parallel Gaussian sub-channels but without water-filling power allocation because the Channel State Information at the Transmitter (CSIT) is not available. In a similar way, if $V_{n}$ denotes the channel dispersion corresponding to the *n*-th time slot, one can see that ${\bar{V}}_{m}≜\frac{1}{G}\sum_{n=(m-1)G+1}^{mG}V_{n}$ yields the channel dispersion for the *m*-th packet. As a result, following the finite-blocklength analysis of [5], channel outage occurs if

$${\bar{R}}_{m}-\sqrt{\frac{{\bar{V}}_{m}}{n}}Q^{-1}\left(\varepsilon \right)<R_{c}.$$

Retransmissions are initiated when a negative acknowledgment (NACK) is received through the feedback channel, prompting the transmitter to schedule the subsequent retransmission. The maximum number of retransmission attempts is limited to $N_{\mathrm{RTX}}$, preventing unbounded delays and resource usage. If decoding fails after the first $N_{\mathrm{RTX}}$ attempts, the packet is declared lost and contributes to the frame error rate (FER) along with the reduction of the system throughput. The transmitter is assumed to have an infinite backlog of packets, ensuring that resources are always utilized and the system operates under a fully loaded traffic scenario.

**Figure 4:**
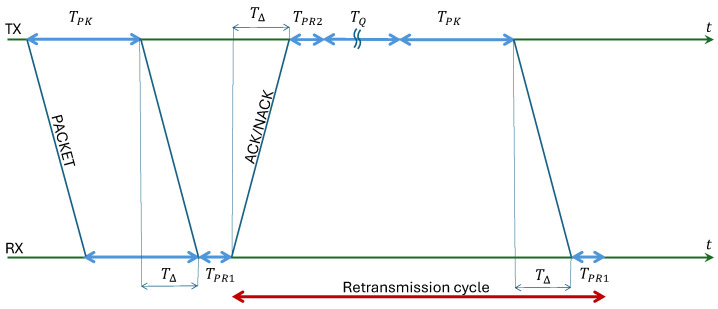
Illustration of transmission delays.

Finally, the total delay of a packet undergoing $n_{\mathrm{RTX}}$ retransmissions accounts for both the packet and feedback transmission delays. Figure 4 illustrates the case of one retransmission. Here, $T_{\mathrm{PK}}$ denotes the packet time; $T_{\Delta }$ the transmission delay; $T_{\mathrm{PR}1}$ the processing time at the receiver; $T_{\mathrm{PR}2}$ the processing time at the transmitter; $T_{Q}$ the queuing time due to rescheduling. As a result, the total delay incurred by one packet is

$$\Delta_{\mathrm{PK}}=T_{\mathrm{PK}}+T_{\Delta }+T_{\mathrm{PR}1}+n_{\mathrm{RTX}}\;\left(2T_{\Delta }+T_{\mathrm{PR}1}+T_{\mathrm{PR}2}+T_{\mathrm{PK}}+T_{Q}\right).$$

The rescheduling time $T_{Q}$ depends on the traffic load and the rescheduling policy, which is modeled as a simple First-In-First-Out (FIFO) queue in which packets are prioritized according to their next minimum rescheduling time (i.e., the earliest time they can be retransmitted after waiting for the ACK). The total processing time $T_{\mathrm{PR}1}+T_{\mathrm{PR}2}$ depends on the hardware and is not considered here. Moreover, the first propagation delay $T_{\Delta }$ is not considered because it is an additive constant for all system configurations. The *end-to-end* packet delay is determined by measuring the time elapsed between the generation and arrival of each packet in the simulation of the queuing process.

### 4.2. Simulation Results

A simulation study has been conducted to evaluate the performance of the system under consideration. This analysis is based on the *power vectors* from [19] for modeling the transmission environment. The primary objective is to investigate how Key Performance Indicators (KPIs), like the Frame Error Rate (FER), the throughput, and the end-to-end delay, are influenced by the packet length and by other critical system parameters. The evaluation is carried out in a scenario reflecting the operational conditions and assumptions relevant to the system design. This approach allows for a thorough understanding of the system’s behavior and provides insights into the interplay between packet size, system configuration, and overall performance.

In the simulations, each PVGeT sample is interpreted as a received power realization $P_{r}\left[n\right]$. We begin by defining the normalized power gain $G_{n}≜P_{r}\left[n\right]/E\left[P_{r}\right]$, and then obtain the fading gain used in (3) as $H_{n}≜\sqrt{G_{n}}$, so that $H_{n}^{2}$ corresponds to the normalized power gain and $E[H^{2}]=1$. This construction is consistent with the SNR definition in (11) and allows the PVGeT power samples to be mapped directly into the parametric model $Y=HX+\sigma_{X}Z$. Given $H_{n}$ and the chosen operating point $\left(\nu_{s},R_{s}\right)$, the corresponding noise variances $\sigma_{0}^{2}$ and $\sigma_{1}^{2}$ follow from (4). Then, the equivalent SNR is obtained from (11), and the instantaneous achievable rate $R_{n}$ from (16).

#### 4.2.1. High-Rate Simulation Results

**Figure 5:**
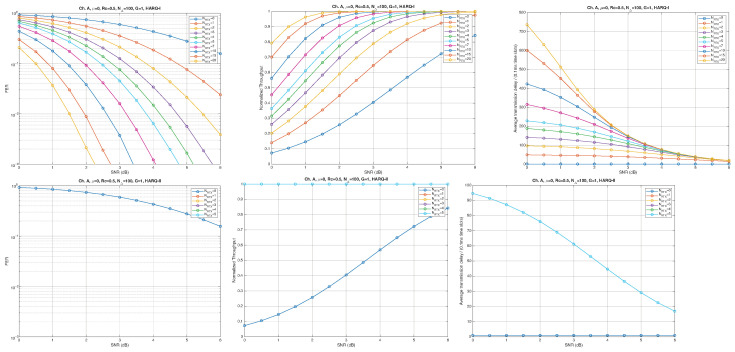
Plots of FER, Throughput, and Delay versus SNR. Channel A, $\mu =0,\;R_{c}=\frac{1}{2},\;N_{\Delta }=100,\;G=1$, and different values of the maximum number of retransmissions allowed $N_{\mathrm{RTX}}$. Upper row: HARQ-I. Lower row: HARQ-II. Some curves in the lower row (HARQ-II) overlap, meaning that from, a certain value of $N_{\mathrm{RTX}}$ on, the performance does not change.

**Figure 6:**
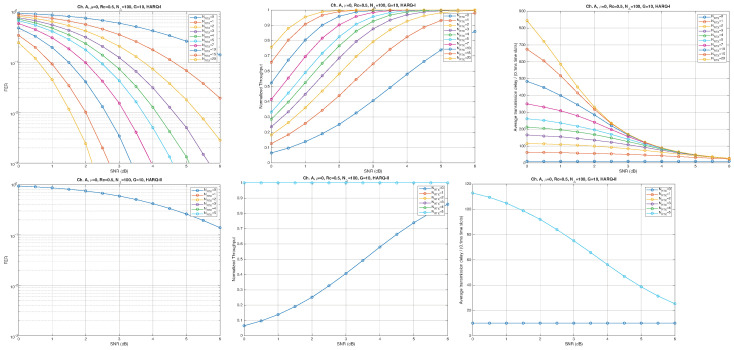
Plots of FER, Throughput, and Delay versus SNR. Channel A, $\mu =0,\;R_{c}=\frac{1}{2},\;N_{\Delta }=100,\;G=10$, and different values of the maximum number of retransmissions allowed $N_{\mathrm{RTX}}$. Upper row: HARQ-I. Lower row: HARQ-II. Some curves in the lower row (HARQ-II) overlap, meaning that from, a certain value of $N_{\mathrm{RTX}}$ on, the performance does not change.

**Figure 7:**
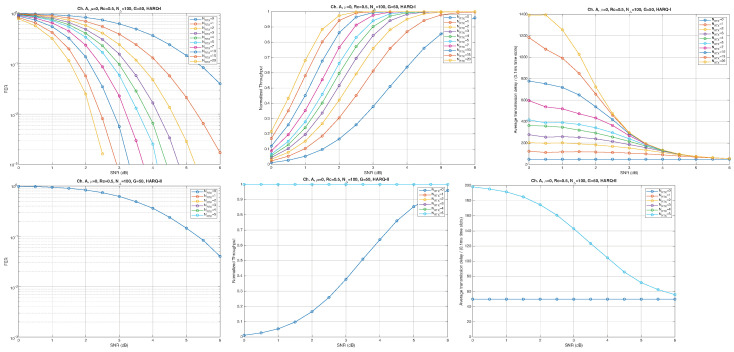
Plots of FER, Throughput, and Delay versus SNR. Channel A, $\mu =0,\;R_{c}=\frac{1}{2},\;N_{\Delta }=100,\;G=50$, and different values of the maximum number of retransmissions allowed $N_{\mathrm{RTX}}$. Upper row: HARQ-I. Lower row: HARQ-II. Some curves in the lower row (HARQ-II) overlap, meaning that from, a certain value of $N_{\mathrm{RTX}}$ on, the performance does not change.

According to the parameters listed in Table 1, simulation results for Channel A, with different packet lengths, μ=0(10Gb/s), HARQ type, and $N_{\mathrm{RTX}}$, are illustrated in Figure 5, Figure 6 and Figure 7 for G=1,10, and 50, respectively. The diagrams are plotted as functions of the SNR and we can notice the following.

**Figure 8:**
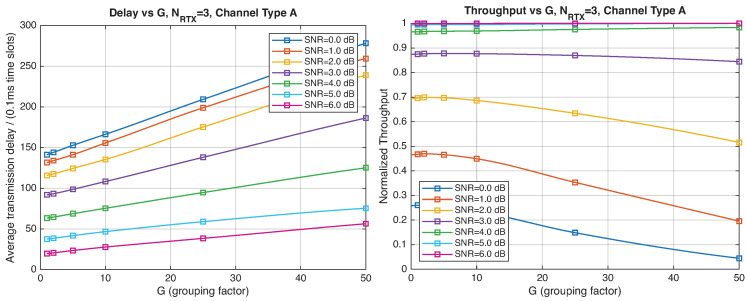
Average transmission delay of successful packets and normalized throughput with HARQ-I versus *G* for $N_{\mathrm{RTX}}=3$ (Channel type A, μ=0, (High rate), $R_{c}=\frac{1}{2},\;N_{\Delta }=100$).

**Figure 9:**
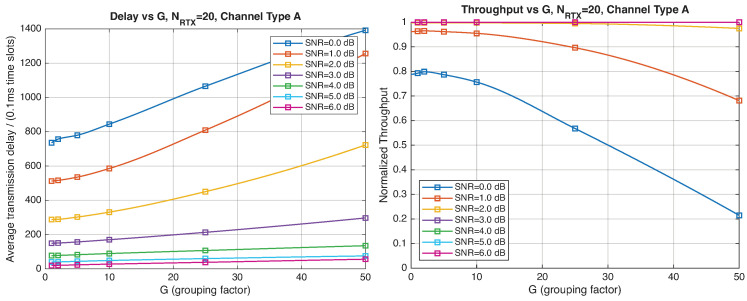
Average transmission delay of successful packets and normalized throughput with HARQ-I versus *G* for $N_{\mathrm{RTX}}=20$ (Channel type A, μ=0, $R_{c}=\frac{1}{2},\;N_{\Delta }=100$).

**Figure 10:**
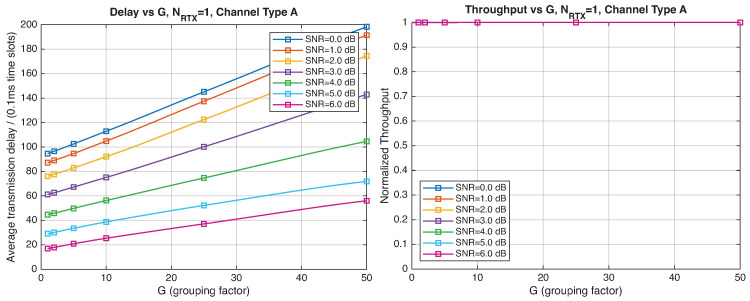
Average transmission delay of successful packets and normalized throughput with HARQ-II versus *G* for $N_{\mathrm{RTX}}=1$ (Channel type A, μ=0, (High rate), $R_{c}=\frac{1}{2},\;N_{\Delta }=100$).

**Figure 11:**
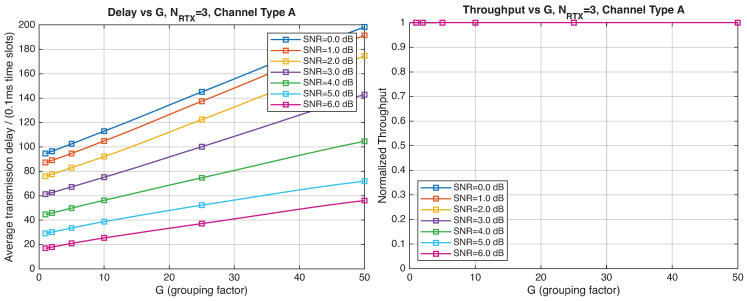
Average transmission delay of successful packets and normalized throughput with HARQ-II versus *G* for $N_{\mathrm{RTX}}=3$ (Channel type A, μ=0, (High rate), $R_{c}=\frac{1}{2},N_{\Delta }=100$).

As expected, the FER decreases as the SNR and the maximum number of HARQ retransmissions, $N_{\mathrm{RTX}}$, increase. It also depends on the packet length *G*, although this effect is less pronounced. Similar considerations apply to the throughput performance. The main performance difference appears in the *average transmission delay*. This is illustrated by the results in Figure 5, Figure 6 and Figure 7 (and some additional results that are not included here for the sake of conciseness), which have been consolidated into Figure 8, Figure 9, Figure 10 and Figure 11 (The lines connecting the markers have been obtained via interpolation).The diagrams show how the delay increases monotonically with *G* so that it is convenient to choose the minimum G=1. The marginal increase in the throughput for small values of G>1 does not justify operating in that region due to the delay penalty. It can also be observed that the primary advantage of HARQ-II over HARQ-I lies in the throughput, whereas the delay improvement is comparatively modest. Similar considerations hold for other channel types and different values of the maximum number of allowed retransmissions $N_{\mathrm{RTX}}$.

#### 4.2.2. Low-Rate Simulation Results

This section extends the analysis reported in Section 4.2.1 by including simulation results for Channel A at a lower bit rate, μ=13(1.22Mb/s). As stated before, the results plot the average delay and throughput versus the packet length factor *G* and emphasize the dependence of the two KPIs on this design parameter.

**Figure 12:**
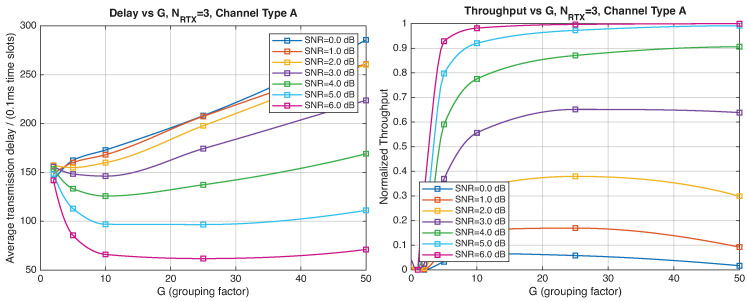
Average transmission delay of successful packets and normalized throughput with HARQ-I versus *G* for $N_{\mathrm{RTX}}=3$ (Channel type A, μ=13, (Low rate), $R_{c}=\frac{1}{2},\;N_{\Delta }=100$).

**Figure 13:**
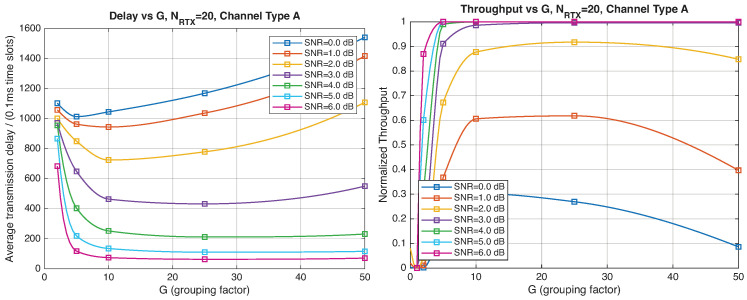
Average transmission delay of successful packets and normalized throughput with HARQ-I versus *G* for $N_{\mathrm{RTX}}=20$ (Channel type A, μ=13, (Low rate), $R_{c}=\frac{1}{2},\;N_{\Delta }=100$).

**Figure 14:**
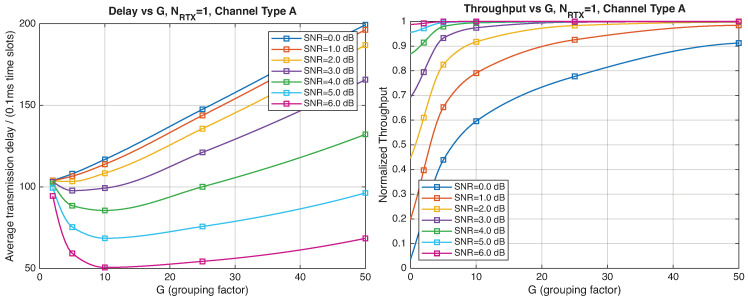
Average transmission delay of successful packets and normalized throughput with HARQ-II versus *G* for $N_{\mathrm{RTX}}=1$ (Channel type A, μ=13, (Low rate), $R_{c}=\frac{1}{2},\;N_{\Delta }=100$).

**Figure 15:**
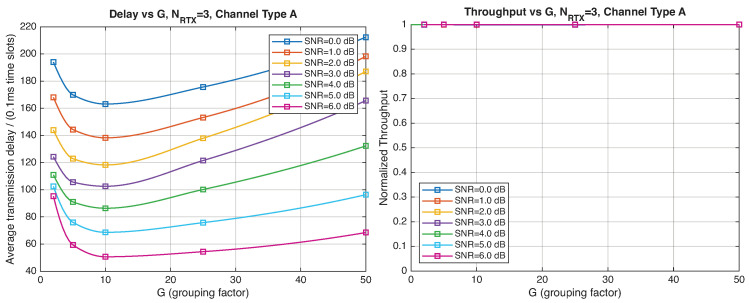
Average transmission delay of successful packets and normalized throughput with HARQ-II versus *G* for $N_{\mathrm{RTX}}=3$ (Channel type A, μ=13, (Low rate), $R_{c}=\frac{1}{2},\;N_{\Delta }=100$).

Figure 12, Figure 13, Figure 14 and Figure 15 show the impact of the packet length *G* on the average delay and throughput, respectively, for several values of the SNR and HARQ-I. It is difficult to provide a uniform interpretation of these results because the KPI behavior is strongly dependent on both *G* and the SNR. As far as the delay is concerned, in most cases (except Figure 12 for low SNR) there is an optimum *G* that minimizes the average delay.

If the SNR is sufficiently low, the average delay grows monotonically with *G* (Figure 12), while above a certain threshold it exhibits an optimum value. The throughput behavior is similar. It grows monotonically with *G* if the SNR is above a certain threshold but it has a maximum below that threshold. This is due to the fact that the finite-blocklength analysis entails a rate degradation depending on *G*. On one hand, increasing the grouping factor increases the average delay because of the longer packets but, at the same time, increases the outage probability, so that, since the average delay is determined over the fewer successful packets, one may observe an improvement in certain conditions. The throughput behavior depends on the SNR, as well. Above a certain threshold, it increases with *G*, while below that threshold it starts increasing and then it decreases so that there is an optimum value. This behavior was barely noticeable in the high-rate simulation results because of the more limited impact of the channel dispersion on the achievable rate (due to the fact that the codewords are shorter in this case). These effects are less noticeable when we consider HARQ-II. In particular, the throughput increases with *G* in all cases considered (Figure 14 and Figure 15). There is still an optimum *G* for the average transmission delay. It can also be noticed that setting $N_{\mathrm{RTX}}=3$ is better than 1 either in terms of average delay and throughput.

#### 4.2.3. Very Low-SNR Simulation Scenario

**Figure 16:**
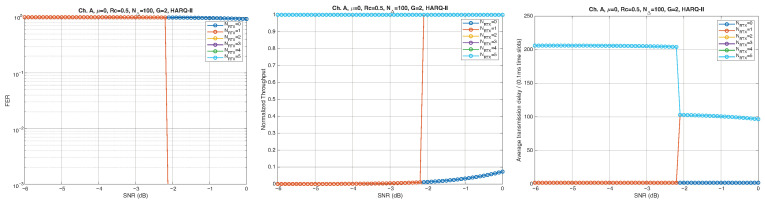
Plots of FER, throughput, and delay versus SNR. Channel A, μ=0, $R_{c}=\frac{1}{2},\;N_{\Delta }=100$, *G* = 2. Protocol: HARQ-II.

**Figure 17:**
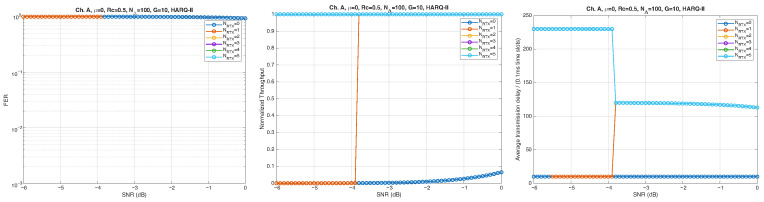
Plots of FER, throughput, and delay versus SNR. Channel A, μ=0, $R_{c}=\frac{1}{2},\;N_{\Delta }=100$, *G* = 10. Protocol: HARQ-II.

**Figure 18:**
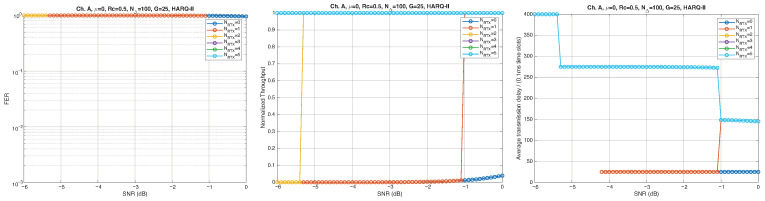
Plots of FER, throughput, and delay versus SNR. Channel A, μ=0, $R_{c}=\frac{1}{2},\;N_{\Delta }=100$, *G* = 25. Protocol: HARQ-II.

**Figure 19:**
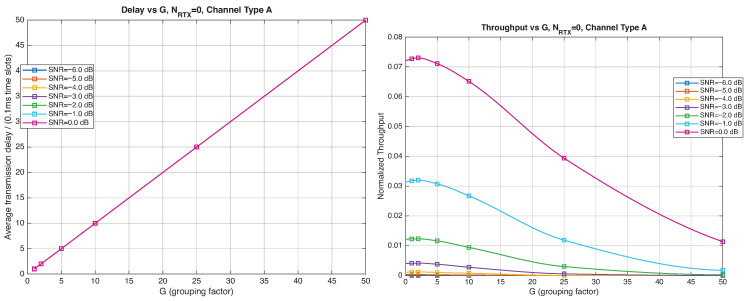
Average transmission delay of successful packets and normalized throughput with HARQ-II versus *G* for $N_{\mathrm{RTX}}=0$ (Channel type A, μ=0, $R_{c}=\frac{1}{2},\;N_{\Delta }=100$, very low SNR).

**Figure 20:**
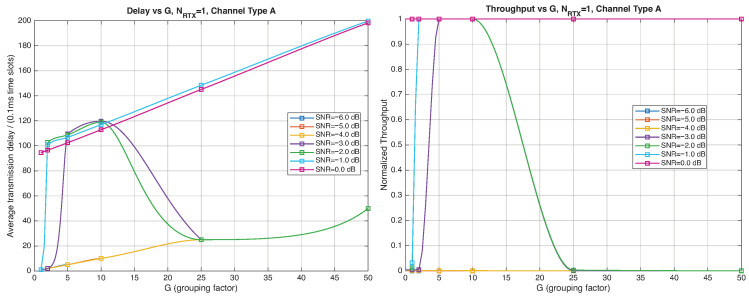
Average transmission delay of successful packets and normalized throughput with HARQ-II versus *G* for $N_{\mathrm{RTX}}=1$ (Channel type A, μ=0, $R_{c}=\frac{1}{2},\;N_{\Delta }=100$, very low SNR).

**Figure 21:**
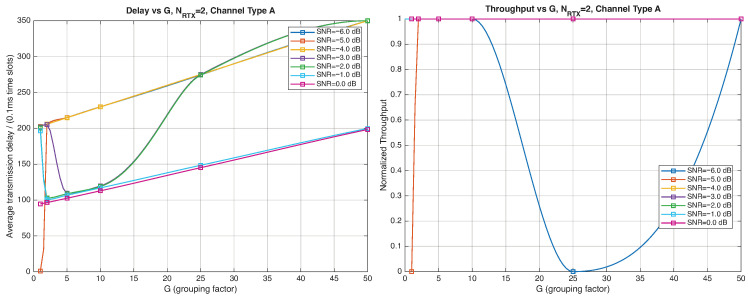
Average transmission delay of successful packets and normalized throughput with HARQ-II versus *G* for $N_{\mathrm{RTX}}=2$ (Channel type A, μ=0, $R_{c}=\frac{1}{2},\;N_{\Delta }=100$, very low SNR).

**Figure 22:**
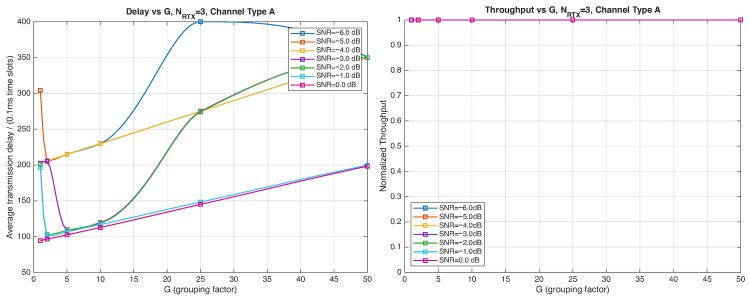
Average transmission delay of successful packets and normalized throughput with HARQ-II versus *G* for $N_{\mathrm{RTX}}=3$ (Channel type A, μ=0, $R_{c}=\frac{1}{2},\;N_{\Delta }=100$, very low SNR).

This section presents simulation results in the very low SNR regime. The analysis focuses on the HARQ-II protocol (since HARQ-I is unable to sustain such SNR levels) with Channel A and μ=0. Figure 16, Figure 17 and Figure 18 report the FER, normalized throughput, and average delay for an SNR range between −3 and 0 dB. Sharp performance transitions are observed at different SNR thresholds for different values of *G*, consistently across the evaluated KPIs. Figure 19, Figure 20, Figure 21 and Figure 22 illustrate the dependence of throughput and delay on the grouping factor *G* for $N_{\mathrm{RTX}}\in 0,1,2,3$. When retransmissions are not allowed ($N_{\mathrm{RTX}}=0$), the normalized throughput reaches its maximum at G=2 but remains extremely low throughout the considered SNR range. Allowing a single retransmission ($N_{\mathrm{RTX}}=1$) significantly improves robustness: for SNR values above −3 dB, the throughput attains its maximum for 5≤G≤10, with a delay only slightly exceeding the round-trip transmission time. However, at lower SNRs, one retransmission is insufficient and increasing the maximum number of retransmissions to $N_{\mathrm{RTX}}=2$ becomes necessary, as shown in Figure 21.

## 5. Conclusions

This paper investigated the performance limits of HARQ-enabled free-space optical communication systems employing OOK modulation and operating under turbulence-induced fading. By combining information-theoretic analyses and extensive simulations based on realistic LEO downlink power vectors, we have characterized the asymptotic finite-blocklength behavior and quantified the impact of packet length and retransmission policies on the main KPIs, namely the FER, the throughput, and the end-to-end delay.

From the analysis of the OOK channel, we derived the achievable rate and the dispersion, which characterize the fundamental transmission limits. The numerical results highlight that the channel dispersion reaches its maximum when the capacity is approximately one half. This implies that finite-blocklength penalties become most pronounced around the coding rates typically adopted in practice. Therefore, HARQ operation in this regime may be more sensitive to turbulence fluctuations and feedback delays.

Simulation results confirm that HARQ-II provides a substantial throughput advantage over HARQ-I across the considered scenarios, while its delay benefits remain more limited. The improved throughput stems from the incremental redundancy gain, which enables HARQ-II to limit the outage probabilities at comparable SNR values. However, the average delay reduction is less significant because the queuing and feedback delays are dominant. Furthermore, the study of the packet length parameter *G* shows that, in the high-rate case, although slightly higher throughput may be achieved for G>1, the gain is marginal and accompanied by a monotonic increase in average delay, making G=1 the most convenient choice. A different situation occurs in the low-rate case where an optimum *G* exists, minimizing the average delay and maximizing the throughput. The low-SNR analysis shows that HARQ-II remains effective even in very challenging operating conditions, with performance strongly influenced by *G* and $N_{\mathrm{RTX}}$. These results underline the importance of proper parameter tuning to maintain throughput and delay efficiency near the system’s reliability limits.

Overall, this analysis provides design guidelines for HARQ-assisted OOK FSO links, highlighting the importance of a proper choice of the packet length, careful selection of retransmission limits, and the role of finite-blocklength effects in determining reliable and low-latency performance in next-generation terrestrial and space optical networks. 

## Figures and Tables

**Table 1:** Typical HARQ simulation parameters.

Parameter	Value
Channel types	A, B, …, H
Rate parameter	$\mu =\left\{\begin{array}{cc} 0 & \left(10.0\;\mathrm{Gb}/s\right)\\ 7 & \left(78.1\;\mathrm{Mb}/s\right)\\ 13 & \left(1.22\;\mathrm{Mb}/s\right) \end{array}\right.$
N. of symbols per slot	$10^{6}2^{-\mu }=\left\{\begin{array}{cc} 10^{6} & \mu =0\\ \approx 7813 & \mu =7\\ \approx 122 & \mu =13 \end{array}\right.$
Round trip delay	10 ms, corresponding to
	$N_{\Delta }=100$ (conservative)
Code rate	$R_{c}=\frac{1}{2}$
HARQ scheme	HARQ-I and HARQ-II

## Data Availability

The original contributions presented in this study are included in the article. Further inquiries can be directed to the corresponding author.

## References

[1] Khalighi M.A., Uysal M. (2014). Survey on Free Space Optical Communication: A Communication Theory Perspective. IEEE Commun. Surv. Tutor..

[2] Uysal M., Capsoni C., Ghassemlooy Z., Boucouvalas A., Udvary E. (2016). Optical Wireless Communications: An Emerging Technology.

[3] Yao Y., Xiao W., Miao P., Chen G., Yang H., Chae C.B., Wong K.K. (2025). UAV-Relay-Aided Secure Maritime Networks Coexisting with Satellite Networks: Robust Beamforming and Trajectory Optimization. IEEE Trans. Wirel. Commun..

[4] Le H.D., Pham A.T. (2022). On the Design of FSO-Based Satellite Systems Using Incremental Redundancy Hybrid ARQ Protocols with Rate Adaptation. IEEE Trans. Veh. Technol..

[5] Polyanskiy Y., Poor H.V., Verdú S. (2010). Channel Coding Rate in the Finite Blocklength Regime. IEEE Trans. Inf. Theory.

[6] Polyanskiy Y., Wu Y. (2025). Information Theory: From Coding to Learning.

[7] Makki B., Svensson T., Caire G., Zorzi M. (2019). Fast HARQ over Finite Blocklength Codes: A Technique for Low-Latency Reliable Communication. IEEE Trans. Wirel. Commun..

[8] Kaushal H., Kaddoum G. (2017). Optical Communication in Space: Challenges and Mitigation Techniques. IEEE Commun. Surv. Tutor..

[9] Majumdar A.K., Ricklin J.C. (2010). Free-Space Laser Communications: Principles and Advances.

[10] Willebrand H., Ghuman B.S. (2002). Free Space Optics: Enabling Optical Connectivity in Today’s Networks.

[11] Bouchet O., Sizun H., Boisrobert C., De Fornel F. (2010). Free-Space Optics: Propagation and Communication.

[12] Majumdar A.K. (2014). Advanced Free Space Optics (FSO): A Systems Approach.

[13] Andrews L.C., Phillips R.L., Young C.Y. (2000). Scintillation model for a satellite communication link at large zenith angles. Opt. Eng..

[14] Andrews L.C., Phillips R.L., Hopen C.Y. (2001). Laser Beam Scintillation with Applications.

[15] Issaid C.B., Alouini M.S. (2019). Level crossing rate and average outage duration of free space optical links. IEEE Trans. Commun..

[16] Giggenbach D., Henniger H. (2008). Fading-loss assessment in atmospheric free-space optical communication links with on-off keying. Opt. Eng..

[17] Moll F. Experimental analysis of channel coherence time and fading behavior in the LEO-ground link. Proceedings of the International Conference on Space Optical Systems and Applications.

[18] Giggenbach D., Parthasarathy S., Shrestha A., Moll F., Mata-Calvo R. Power vector generation tool for free-space optical links—PVGeT. Proceedings of the 2017 IEEE International Conference on Space Optical Systems and Applications (ICSOS).

[19] Giggenbach D., Shrestha A., Moll F., Fuchs C., Saucke K. Reference power vectors for the optical leo downlink channel. Proceedings of the 2019 IEEE International Conference on Space Optical Systems and Applications (ICSOS).

[20] Li D., Li P., Zhao J., Liang J., Liu J., Liu G., Lei Y., Liu W., Deng J., Liu F. (2024). Ground-to-UAV sub-terahertz channel measurement and modeling. Opt. Express.

[21] Kiasaleh K. (2010). Hybrid ARQ for FSO Communications through Turbulent Atmosphere. IEEE Commun. Lett..

[22] Aghajanzadeh S.M., Uysal M. (2012). Information Theoretic Analysis of Hybrid-ARQ Protocols in Coherent Free-Space Optical Systems. IEEE Trans. Commun..

[23] Zedini E., Chelli A., Alouini M.S. (2014). On the Performance Analysis of Hybrid ARQ with Incremental Redundancy and with Code Combining over Free-Space Optical Channels with Pointing Errors. IEEE Photonics J..

[24] Makki B., Svensson T., Eriksson T., Alouini M.S. (2016). On the Performance of RF-FSO Links with and without Hybrid ARQ. IEEE Trans. Wirel. Commun..

[25] Touati A., Hasna M.O., Touati F. HARQ Performance over FSO Channels with Atmospheric Fading and Pointing Errors. Proceedings of the 2018 14th International Wireless Communications & Mobile Computing Conference (IWCMC).

[26] Verma G.D., Mathur A. (2021). Performance Improvement of FSO Communication Systems Using Hybrid-ARQ Protocols. Appl. Opt..

[27] Schieler C.M., Garg A.S., Bilyeu B.C., Wang J.P., Robinson B.S. Demonstration of reliable high-rate optical communication over an atmospheric link using ARQ. Proceedings of the 2019 IEEE International Conference on Space Optical Systems and Applications (ICSOS).

[28] Poulenard S., Gadat B., Chouteau J., Anfray T., Poulliat C., Jego C., Hartmann O., Artaud G., Meric H. Forward error correcting code for high data rate LEO satellite optical downlinks. Proceedings of the International Conference on Space Optics—ICSO 2018.

[29] Mazzali N., Arapoglou P.D. Channel interleaver dimensioning for optical LEO direct-to-earth systems. Proceedings of the 2020 10th Advanced Satellite Multimedia Systems Conference and the 16th Signal Processing for Space Communications Workshop (ASMS/SPSC).

[30] Artaud G., Chouteau J.F., Barthe L., Gadat B., Anfray T., Poulenard S., Thomas A.D., Quentel A. Design and validation of a new coding and synchronization layer for space optical communications. Proceedings of the International Conference on Space Optics—ICSO 2022.

[31] Arrieta D.R., Almonacil S., Conan J.M., Paillier L., Dutisseuil E., Bigo S., Renaudier J., Boddeda R. (2023). Proof-of-concept real-time implementation of interleavers for optical satellite links. J. Light. Technol..

[32] Nguyen D.T., Park Y. (2019). Performance analysis of interleaved LDPC for optical satellite communications. Opt. Commun..

[33] Matuz B., Zahr A., Sauter A. Coherent communications for free space optical low-earth orbit downlinks. Proceedings of the GLOBECOM 2022-2022 IEEE Global Communications Conference.

[34] Yamazoe H., Ohta S., Komatsu H., Suzuki K., Okamoto E., Iwamoto K. Evaluation of the forward error correction format for LEO-ground optical communication using Reed-Solomon product code. Proceedings of the Free-Space Laser Communications XXXIII.

[35] Arapoglou P.D., Colavolpe G., Foggi T., Mazzali N., Vannucci A. (2022). Variable data rate architectures in optical LEO direct-to-earth links: Design aspects and system analysis. J. Light. Technol..

[36] Esmail M.A., Fathallah H., Alouini M.S. Analysis of fog effects on terrestrial Free Space optical communication links. Proceedings of the 2016 IEEE International Conference on Communications Workshops (ICC).

[37] Ojo J., Olaitan J., Ojo O. (2022). Characterization of fog-induced attenuation for optimizing optical propagation links in Nigeria. Results Opt..

[38] (2020). Recommended Standard + Pink Sheets for O3K—Optical Communications Physical Layer.

[39] Montorsi G. (2022). Personal Communication.

[40] Hamkins J., Divsalar D. (2020). Presentation at CCSDS Optical Communications Working Group Monthly Meeting.

[41] Abramowitz M., Stegun I.A. (1970). Handbook of Mathematical Functions.

[42] Lin S., Costello D.J., Miller M.J. (1984). Automatic-repeat-request error-control schemes. IEEE Commun. Mag..

[43] Frenger P., Parkvall S., Dahlman E. Performance Comparison of HARQ with Chase Combining and Incremental Redundancy for HSDPA. Proceedings of the IEEE 54th Vehicular Technology Conference (VTC-Fall 2001).

[44] Hamzaoui R., Stanković V., Xiong Z., Ramchandran K., Puri R., Majumdar A., Chou J. (2009). Chapter 3.4—Channel Protection Fundamentals. Communications Engineering Desk Reference.

[45] Dahlman E., Parkvall S., Sköld J. (2014). Chapter 12—Retransmission Protocols. 4G: LTE/LTE-Advanced for Mobile Broadband.

